# Equity consideration in palliative care policies, programs, and evaluation: an analysis of selected federal and South Australian documents

**DOI:** 10.1186/s12904-022-00997-2

**Published:** 2022-06-16

**Authors:** Sara Javanparast, Julia Anaf, Jennifer Tieman

**Affiliations:** 1 Research Centre for Palliative Care, Death and Dying, College of Nursing and Health Sciences, Flinders Medical Centre, Flinders University, Bedford Park, SA 5042 Australia; 2 College of Medicine and Public Health, Flinders University, Bedford Park, Australia

**Keywords:** Palliative care, Public health approach, Policy, Equity, Australia

## Abstract

**Background:**

Inequity in access to palliative care and symptom relief is one of the greatest disparities in global health care. A public health approach to palliative care is underpinned by the social view of health that puts an emphasis on equity, community engagement and empowerment, a supportive policy environment, and social determinants of health. Consideration of equity in policy is critical so that it can be translated into equitable services. However, the extent to which Australian palliative care policies incorporate equity, and their translation into actual actions have not been extensively examined.

This exploratory study aimed to examine the extent to which Australian federal and South Australian palliative care policies and initiatives incorporate equity, and to identify evidence gaps and research priorities that can inform equity-oriented policies and practices.

**Methods:**

We reviewed 25 federal and South Australian documents relating to palliative care published over the past five years. Documents were publicly available from the Australian Government Department of Health website. We used search filter ‘Palliative care and end of life’ in the Department’s resource webpage to narrow down documents to those with palliative care and end of life in the document title and/or content. The initial list was discussed in the research team to ensure key documents are included. Supplementary to document review, we conducted five key informant interviews in South Australia. Interview participants were people from the policy sector, not-for-profit organisations, a funding body and a community advocacy group in South Australia who had knowledge and experience in palliative care policy, practice and research. Documents and interview transcripts were imported into the NVivo 12 software for coding. Content analysis looked at the frequencies of relevant terms, and then more detailed inductive and deductive thematic analysis was undertaken which was guided by an equity action framework.

**Results:**

Overall, we found incremental steps forward over the past few years in considering equity in Australian palliative care policies. Key themes that emerged from the study were: identifying population groups experiencing poor access to palliative care, strategies to improve access including increased awareness of palliative care, flexible models of care, building workforce capacity, and the need for greater investment in palliative care research and evaluation. Strategies to address systemic barriers as well as social, political and cultural determinants of inequity was less evident in policy documents. There was little evidence of actions to engage and empower communities. Interviews provided insight on key areas of priority for future palliative care research.

**Conclusions:**

Achieving the goal of equity in palliative care for all is complex and multifaceted. It requires strong commitment and actions at policy and government level but also in clinical practice, workforce planning and capacity building, community engagement and research investment to implement and evaluate public health approaches to palliative care.

**Supplementary Information:**

The online version contains supplementary material available at 10.1186/s12904-022-00997-2.

## Background

Health inequity is defined as disparities in health between population groups that are avoidable, unfair and unjust [1]. Unequal access to palliative care and symptom relief is one of the greatest disparities in global health care with only 14% of patients who need palliative care receive it [2]. Equity is about improved access to existing care services including clinical care, but also complementary models of care to address other elements of equity making palliative care an ethical responsibility of health systems [2].

Clinical and specialised palliative care services including symptom management are critical to address patient’s physical distress [3, 4]. However, there has been a growing interest in broader emotional, social and spiritual dimension of distress amongst palliative patients and societal frameworks that support individuals approaching the end of life such as public health, health promoting palliative care and compassionate communities [5, 6]. The history of hospice care goes back to 1950’s when the first modern hospice was established by Dame Cicely Saunders. She introduced the idea of ‘total pain’ which included the physical, emotional, social, and spiritual dimensions of care [7]. In 1999, Kellehear proposed a health promoting palliative care model, underpinned by the Ottawa Charter, which conceptualised an understanding of palliative care in social and political terms not clinical care alone [8]. In 1990, the World Health Organization pioneered the public health model of palliative care by focusing on supportive policy environment, access to essential medications, public and professional education, and implementation of services at all levels of the society [2]. Quality of life and early identification, assessment and treatment of physical, psychosocial and spiritual problems of patients and their families were strongly emphasised [2].

Scholars have described theoretical and essential features of public health palliative care through sociological perspectives [9, 10]; examined rationales for applying a public health approach and the ways that such an approach can support local service planning and delivery [11]; reviewed impacts on community actions [12]; mapped activities and programs that could be classified as public health palliative care [13]; and recommended ways to address barriers to access a high quality and integrated palliative care [14].

The underpinning value in a public health approach in palliative care is the social view of health that puts an emphasis on equity and inclusion, community engagement and empowerment, a supportive policy environment, advocacy, and attention to the wider determinants of health [15]. Such approaches strengthen and complement clinical and medical services by taking the person’s dignity and capability into consideration [16]. It has clear implications for organisations and services and the broader society, and a ‘way of being in the professional world’ [17].

Some countries implemented initiatives to strengthen public health approaches in palliative care at policy and practice levels. In Scotland public health and palliative care experts engaged in complex policy development processes to improve equity in access to palliative care for communities [18]. In 2015, a UK review of equity in the provision of palliative care and its economic implications demonstrated wide service gaps, poor coordination and integration, and an urgent need for non-clinical palliative care [19]. Equity impact assessment of palliative care policies has been conducted in some countries such as the UK to identify equity needs and gaps, monitor inclusion progress, and to ensure that policies and initiatives do not inadvertently create inequity [18, 20]. An analysis of palliative care policies in Canada found that although inequity is well-documented, the policy and political contexts hinder the incorporation of public health approaches in palliative care [21].

To date, research on equity in palliative care has mainly focused on identifying priority population groups including Aboriginal and Torres Strait Islander peoples, non-English speaking communities, people living in rural areas, and those with low socioeconomic status, and key barriers in access to professional and clinical services such as poor understanding of palliative care, communication and cultural barriers, distance, and cost [22]. Recognition of socio-demographic disparities across population groups is essential to ensure services are tailored based on the needs of each group [23]. There is however paucity of evidence concerning the social determinants of access to palliative care including cultural acceptability, affordability, and social inclusion [24]. Social determinants of health are integral to achieving health equity with increasing research and evidence on the social determinants of equity in palliative care are being available [25].

In Australia, federal and state/territory palliative care policies and strategies have been developed, updated and evaluated over time. Palliative care services are provided in various settings including general practice, acute hospital, residential aged care and patient’s home [26]. Specialist palliative care services are provided in inpatient consulting services, hospices and community-based specialist services [26]. Each state/territory has its own policy/framework and/or range of activities in place to improve palliative care. In South Australia, palliative care is often provided by general practitioner and other primary health care providers while specialist palliative care team works in a consultative role when referrals needed [27].

Policy context supportive of equity acts as a critical driver for enabling its translation into equity-oriented actions [28]. However, the extent to which Australian palliative care policies incorporate equity, and their translation into actual actions and investments has not been extensively examined. This study is the first, as part of a larger proposed program of research, to explore equity considerations in Australian palliative care policies. We specifically focused on federal and South Australian policy and program documents to:

1. examine the extent to which policies and initiatives incorporate a public health approach including equity and social determinants of health, and their underlying enablers and barriers.
2. identify evidence gaps and research priorities that can inform equity-oriented palliative care policies and practices.

## Methods

### Document review

We conducted a document review including palliative care policies and strategic plans, reports of government-funded programs and initiatives, policy or program evaluation reports and needs assessment reports. We also reviewed the Australian Royal Commission into Aged Care Quality and Safety final report published in 2021. Although the Commission is independent of government, their report and recommendations were deemed to be important in policy and practice directions. Federal and South Australian documents over the last five years were included to cover more recent documents. Due to time and funding constraints, we only included South Australia as an example of jurisdictional policies and initiatives as authors had higher knowledge of the context and better connection to key stakeholders in the state. Although the South Australian strategic plan (2009–2016) was published in 2009, we included the document as it was the latest and the only palliative care strategic plan in South Australia.

We searched the Australian Government Department of Health website where key federal and jurisdictional documents are made publicly available. We used search filter ‘Palliative care and end of life’ in the Department’s resource webpage to narrow down documents to those with palliative care and end of life in the document title and/or in content. The initial list was discussed in the research team to ensure key documents are included.. A total of 25 documents (21 federal and 4 South Australian) documents were found. Table 1 shows the title, year, category and description of each document.

**Table 1:** Name, category and description of documents included in the review

Title	Year	Category	Description	Equity consideration
**National documents**				
1. Royal Commission into Aged Care Report	Feb 2021	Recommendation	The report present findings of the Royal Commission into Aged Care Quality and Safety work and their recommendation for aged care system reform in Australia including palliative and end of life care	Acknowledges the lack of care for those with ‘special needs’; highlights underserved populations e.g. homeless, Indigenous people and those in rural settings. Recommends the right to fair, equitable and non-discriminatory access to palliative and end-of-life care.
2. Evaluation of the End-of-Life Directions for Aged Care Program	Dec 2020	Evaluation Report	The report is a summary of an evaluation of the End-of-Life Directions for Aged Care (ELDAC) implemented from 2017 to 18 to 2019–20	No reference to equity and social determinants of palliative care
3. Budget 2020–21: Aged Care – Greater Choice for At Home Palliative Care	Oct 2020	Government Budget Fact Sheet	A Budget 2020–21 fact sheet is part of a collection on government ageing and aged care budget	The only mention of equity is: ‘addressing people’s need wherever they live’
4. Implementation Plan for the National Palliative Care Strategy 2018	Oct 2020	Policy, strategy or framework	Implementation Plan for the National Palliative Care Strategy provides a guide to improve the implementation of palliative care services in Australia	Focuses on priority population groups and includes action plan to address access issues for these groups
5. Evaluation of the National Palliative Care Projects	March 2020	Evaluation Report	The report presents the findings of the evaluation of the 13 Commonwealth funded palliative care projects. The projects primarily focused on workforce education and training, quality improvement and advance care planning and did not fund palliative care service delivery.	Includes extensive discussion on collaboration but no reference to equity or social determinants of palliative care,
6–16. Exploratory analysis of barriers to palliative care: Summary Policy Paper plus 9 individual reports for: people from culturally and linguistically diverse groups; Aboriginal and Torres Strait Islander peoples; care leavers and people affected by forced adoption; people with disability; people experiencing homelessness; people who identify as lesbian, gay, bisexual, transgender or intersex; people who are incarcerated; refugees; veterans	Jan 2020	Review Report These include one overall summary report as well individual reports for each priority groups.	The summary policy paper and series of nine full reports presents the exploratory analysis of barriers and facilitators to access to high quality palliative care that are common to most or all of 9 under-served populations in Australia.	All documents have an extensive focus on equity and social determinants of palliative care highlighting shared difficulties and more nuanced information for each of the underserved populations
16. National Palliative Care projects - Grant opportunity	June 2019	Grant Opportunity Guideline	The guideline outlines eligibility to apply for national palliative care grants to improve access to high quality palliative care in Australia, and to enhance the quality of palliative care service delivery and provide support for people who are dying, their families and carers	Prioritises projects that ‘Improve the health of targeted populations that experience health inequalities or social disadvantage including those based on gender, culture, age and disability’.
17. The National Palliative Care Strategy 2018	Feb 2019	Policy, strategy or framework	National Palliative Care Strategy 2018 is the latest national policy in Australia and was developed following the 2016 evaluation of the 2010 national strategy. The strategy outlines government’s higher-level strategies for palliative care in Australia	Focuses on underserved populations and structural barriers of access and choice
18. Evaluation Plan for the Greater Choice for At Home Palliative Care	Jan 2019	Evaluation Plan Report	This evaluation plan sets out the approach for the evaluation of the Greater Choice for At Home Palliative Care Measure.	Mentions engagement with underserved populations. A strong focus on equity with ‘equity’ being one of the evaluation reporting domain.
19. Greater Choice for At Home Palliative Care	Nov 2018	Project Report	This report summarises palliative care activities coordinated through 11 Primary Health Networks (PHNs) in Australia. PHNs are Australian regional primary health care organisations	Only one of the participating PHNs focused on underserved populations – CALD and refugees. No mention of equity or social determinants of palliative care
20. Evaluation of the National Palliative Care Strategy 2010	Sep 2016	Evaluation Report	The report presents the findings of the evaluation of the 2010 National palliative Care Strategy. The aim of the evaluation was to determine how relevant and useful the strategy is for palliative care in Australia.	Reports that lack of attention to underserved populations and notes a disparity between urban and rural settings. Highlights the need for greater focus on underserved populations
21. Palliative care in Australia	May 2016	Government response to inquiry	This report details Health’s response of the Australian Government to the Senate Community Affairs References Committee report: Palliative Care in Australia.	Provides recommendations to identify potential gaps and improve palliative care for people with disabilities and Indigenous people
**South Australian documents**				
22. Palliative Care Strategic Framework 2021–2026 (draft)	June 2021	Policy, strategy or framework	The strategic framework has been developed in recognition of a need for a clear and consistent narrative that identifies priorities and guides collective efforts to improve palliative care services in South Australia	Focuses on each of the underserved populations and notes the need for greater collaboration
23. Palliative Care 2020 Grants Program Project Showcase	June 2021	Report	The Palliative Care 2020 Grants Program provided $1.4 million in funding to 16 non-government organisations to undertake 17 projects. The projects focus on improving services for those in our community who were identified with greater needs in the 2019 state-wide assessment of palliative care needs.	Funds project with a focus on those with greater needs (6 out of of 17 projects)
24. Palliative care needs in South Australia	Oct 2019	Review Report	The report presents palliative care needs in different settings and for different population groups in South Australia	Acknowledges people with complex needs, disabilities and Indigenous people, CALD communities, children and rural and remote.
25. Palliative Care Services Plan 2009–2016	May 2009	Policy, strategy or framework	The paper outlines the South Australian Government’s plan to expand and reshape services, in light of increasing demand for end-of-life care across the health system.	Notes that people need help regardless of locationaand focuses on improving access and equity, discusses the social gradient.

We reviewed selected policy and program documents to capture:

- Ways in which equity is defined (e.g., improved assess to clinical services, service availability for priority population groups, social determinants of health).

- The extent to which recommended strategies and actions are equity-oriented (e.g., intersectoral collaboration, community engagement, and activities to address sociocultural determinants)

- The extent to which evaluations incorporate equity domains and measures
- Evidence of investment in equity-oriented initiatives, projects and research
- Range, purpose and strength of equity-related evidence used to inform policy or practice.

Our review was informed by the equity action framework developed by Freeman et al. for regional primary health care organisations in Australia [24]. This framework acknowledges the importance of equity as an organisational goal, and collection of health equity data but also considers effective strategies to address health inequities, including equity impact evaluation of initiatives, community participation and engagement with communities affected by health inequities, addressing social determinants of health inequities, and intersectoral collaboration [29]. The framework highlights the need for transformation of power relationships underpinning inequities, and more equitable distribution of social, political and cultural determinants of health [29].

### Key informant interviews

Supplementary to document review, we conducted five individual interviews with people from the policy sector, not-for-profit organisations, a funding body and community advocacy group who had knowledge and experience in palliative care in South Australia. Interviews aimed to gain additional insights from key actors with diverse experience in different aspects of palliative care. Using the lead author’s extensive experience and knowledge of palliative care context and players in South Australia, we used purposeful sampling to recruit key informants for interview. An interview guide was developed and discussed in the research team covering participants’ views on equity in palliative care, examples of equity-oriented policy or programs, and the role of evidence and research priorities to inform palliative care policy and practice in the future (See supplementary file). Interviews took approximately one hour and were conducted virtually in October 2021 by the second author, an experienced qualitative researcher. Consent to participate and record and transcribe the interviews was obtained from respondents.

### Analysis

Documents were imported into the NVivo 12 software for coding and analysis. We developed an initial coding framework guided by the equity action framework [24]. These included codes in relation to equity as a policy goal and objective, equity in access, availability and affordability of palliative care services, intersectoral collaboration, community engagement and social determinants of palliative care. Additional coded were added inductively during review process. Examples include equity-focused funding allocation and equity related evidence to inform policy. Inductive and deductive thematic analysis was used to identify and categorise key themes. Content analysis looked at the frequencies of relevant terms, and then more detailed thematic analysis was undertaken. For interview transcripts we added specific codes to the existing framework on enablers and barriers to equity-focused policy and practice, evidence gap and research priorities. Data from interviews was used to complement document review through exploration of participant views on equity and its consideration in current policy context as well as examples of equity-oriented palliative care interventions and investments.

Ethics approval (ID 4627) for individual interviews was granted from the Flinders University Social and Behavioural Research Ethics Committee.

## Results

We identified six key themes related to ways in which equity was reflected in palliative care policies and initiatives, and evidence gaps and research priorities to inform equity-oriented palliative care policy and practice. Key findings are presented below:

### Identifying population groups experiencing inequity in access to palliative care

There was evidence of a shift towards equity consideration in national and state policies and initiatives. The 2016 evaluation of the 2010 National Palliative Care Strategy reported a lack of focus on palliative care needs of priority population groups, and inconsistent and inequitable public investment and service provision. The 2018 National Palliative Care Strategy and Implementation Plan acknowledged that ‘*palliative care is not equally available to all people across Australia, for reasons of geography, awareness, economics, workforce, and accessibility*’ [30].

Equity consideration was also reflected in South Australian documents. The state Palliative Care Needs Assessment highlighted inequity in access to palliative care: *‘Some populations have limited or no access to services and where available, services may be inappropriate to their physical, social, cultural and spiritual needs*’ [31]. The SA Palliative Care Plan 2009–2016 also recognised *‘a social gradient where the needs of disadvantaged communities require particular attention to ensure equity of access and comparable care outcomes’* [32, 33].

However, identifying populations experiencing inequity and gathering evidence on access barriers was the most common area of consideration, and found in 17 of the 25 documents. The 2018 National Palliative Care Strategy identified people who are Aboriginal and Torres Strait Islander, culturally and linguistically diverse (CALD), lesbian, gay, bi-sexual, trangender or intersex (LGBTI), living in rural and remote areas, living with disabilities, experiencing homelessness, and incarcerated as priority groups who face structural or systemic barriers to accessing quality palliative care [30]. A series of exploratory analysis reports commissioned by the Australian federal Department of Health focused on nine population groups that are prioritised in the Aged Care Act [34]. These reports had the most extensive focus on equity: ‘*This is necessary to ensure that underserved populations do not get ‘left behind’ … Otherwise, these initiatives may service to widen the gaps between under-served groups and the rest of the population’* [35]. The report on access barriers for Aboriginal and Torres Strait Islander people, for example, highlights the issues around racism, cultural stereotyping and difficulty in accommodating cultural practices in palliative care settings, fear or mistrust of western medicine, and language and communication issues [36]. To address inequity, greater engagement between services and communities, and recruitment of Aboriginal and Torres Strait Islander staff were recommended [36]. SA documents followed the national policies in identifying similar equity groups and the need to improve access to palliative care services for these groups [31, 32].

### Strategies to improve access and quality of palliative care

The aspiration for improved equity in access to existing palliative care services for all was a common theme. Fourteen out of 25 documents proposed strategies to improve equity for priority population groups. Examples included increased awareness and understanding of palliative care services, flexible models of palliative care, employing members of diverse communities in different roles to provide palliative care, increased funding and resources, building care providers’ capability, and improving research and evaluation.

The 2018 Exploratory Analysis on barriers to care stated ‘*It may be most important to shift from providing special care to people belonging to under-served populations, to instead providing inclusive care to all people, including those from these groups’* [35]. Adopting a universalist perspective was identified in the national 2020–21 Budget for the Greater Choice for At Home Palliative Care to *‘support access to end-of-life care for Australians, regardless of where they live*’ [37]. The latest SA Draft Palliative Care Framework (2021–2026) took a person-centred approach so that ‘*all South Australians have access to and receive the best possible palliative care that places the person at the centre of care and supports them to live and die well in accordance with their individual needs, wishes, values and preferences’* [33].

The Royal Commission into the Aged Care Quality and Safety Report (2021) recommended strategies in aged care settings as ‘*a right to fair, equitable and non-discriminatory access to palliative and end-of-life care, improved access to specialist services and requirements for regular staff training. Urgent consideration should also be given to how palliative care is reflected in the Aged Care Quality Standards’* [38].

In five documents, innovative models of care were encouraged, funded, or implemented. Examples included the use of telehealth or specifically-designed ‘apps’ to support palliative care knowledge in communities that may be poorly resourced or isolated [35], care models to meet the needs of Indigenous populations living with end-stage disease by getting people back to their country area, placing cultural priorities at the centre of care through ‘deep caring and listening’, and providing basic supplies and extra care or support workers [36]. A South Australian funded project in 2020 ‘In-Home Hospice Care’ provided training for volunteers and free compassionate family-centred care at home at any time. This project resulted in an improvement in addressing physical, emotional, social, cultural and spiritual needs of people dying at home, and their families [39].

### Intersectoral collaboration and community engagement

Thirteen out of 25 documents acknowledged intersectoral collaboration, with 10 documents proposing actions including forging links with community organisations, social care services, the Aboriginal community-controlled sector, and a better connection between health systems, and disability and aged care sectors to improve care pathways. Collaboration was a goal in the 2018 National Palliative Care Strategy, emphasising working across primary health care, hospitals, Aboriginal services, and private and not-for-profit providers to reduce duplication and share information and innovation [30]. A public health approach involving multiple sectors was particularly reflected in a series of documents that explored access barriers in nine priority population groups stating: ‘*networks, partnerships and collaborations are vital to supporting all Australians in the promotion and provision of quality palliative care. Such collaborations can help to break down the silos in current service delivery and to raising community awareness through to providing person centred care. They support a ‘no wrong door’ approach …* [35].

The South Australian documents also had an emphasis on *‘whole of system effort’* and ‘*identification of service models that improve intersectoral collaboration*’ and *‘improving patient data sharing and addressing funding barriers that inhibit cross sectoral collaboration*’ [33, 39].

Despite examples of activities on collaborations occurring within health system for example between primary health provider and palliative care specialists and to some extent between health and aged care settings, we found only two examples demonstrating collaboration with organisations outside health system particularly social sectors and community-based organisations. One was collaboration between a regional primary health care organisation with six aged care centres to establish an inter-sectoral collaboration group to deliver palliative care quality improvement packages across aged care facilities [37]. A South Australian-funded project also aimed to improve collaboration between academics, specialist palliative care clinicians and community organisations to explore community perspectives on palliative care, developing community resources, and enacting cultural and spiritual practices in hospitals [39]. Collaboration with non-health sectors was a major gap in policy documents.

Involving individuals and their families/carers in the design, implementation and evaluation of palliative care services was prioritised in the 2018 National Palliative Care Strategy. However, explicit evidence on community engagement in the design and implementation of palliative care services was only demonstrated in three documents. Examples included engagement with Aboriginal communities, as part of the reference group, to oversee the development of a palliative care training package, and engagement with people from different ethnic backgrounds to develop community resources and documentary films regarding palliative care, death and dying [39]. Community empowerment is the process of enabling communities to increase control over their lives by gaining control over the decisions and factors that shape their lives [40]. This involves ceding or sharing power more equitably. However, there was a lack of clarity about the role of community empowerment and advocacy towards equitable palliative care, including whose voices are privileged when determining community needs.

### Actions to address social determinants of equity in palliative care

Despite an acknowledgement of social determinants in 19 of 25 documents and presenting evidence on non-health related issues hindering access to palliative care in four of 25 documents, the review found no evidence of actions to address social determinants of health. For example, documents providing an exploratory analysis of barriers to palliative care across nine under-served populations made explicit references to social determinants of palliative care. These included education, income, cultural stereotyping and language barriers, poor health literacy, religious determinants, stigma and institutional racism, intergenerational trauma, housing and location as social determinants to be addressed to provide equitable and quality palliative care [35]. These documents noted that some groups were ‘doubly vulnerable’, due to palliative care contextualised by the experience of deficits in social determinants of health. They promoted a public health approach to palliative care and a move from a specifically clinical approach towards community development, and social research to address ‘organisational, structural and cultural considerations required for systemic and sustainable change in end-of-life practice’ [35].

### Equity-focused research and evaluation

Despite a general focus on equity, the implementation of proposed strategies and evaluation of their impact on access and quality of palliative care was less apparent. A major evaluation report assessing the outcomes of 13 national palliative care projects between 2017 and 2020, lacked any questions and indicators to measure the extent to which these projects, individually or collectively, impacted on equity [41]. Only one document (2019 Evaluation Plan for the Greater Choice for At Home Palliative Care Evaluation) [42] included equity as an evaluation domain to assess equity of program outcomes for certain population groups. None of the evaluation reports included measures to evaluate the process or outcomes in relation to addressing social determinants of palliative care [41–44].

### Evidence gaps and research priorities in equity and palliative care

The need for evidence-based practice was acknowledged in 14 documents. Types of evidence recommended were multi-faceted, included information based on consultations, benchmarking, performance indicators and datasets. Nevertheless, specific evidence gaps on equity-focused policy and practice and research priorities were not identified in documents [33, 39, 45]. While a series of reports commissioned by the Australian Government gathered extensive evidence concerning the barriers to access and equity for nine under-served groups, the way evidence was used to inform specific policy area, strategy or initiative was less evident. Similarly, a number of South Australian funded projects helped to generate evidence, one of which consulted Indigenous expertise in respect of community, industry and workforce to oversee and guide training development. South Australia’s Palliative Care Strategic Framework explicitly emphasised continued support to palliative care research and translation of research into practice with no mention of priority areas and evidence gaps. Qualitative interviews with five key informants in South Australia were used to supplement document reviews and to provide further information on evidence gaps and research priorities around equity and palliative care.

### Key informant perspectives

Interviews provided supplementary data with respondents providing a range of key insights to augment the document analysis. Respondents were specifically questioned about their views on how policies and initiatives have been able to address inequity; their views on key enablers and barriers to achieving equity-focused palliative care; and the evidence gaps and research priorities that could facilitate achieving equity. Two categories of findings emerged from interviews that are highlighted across Tables 2 and 3. These were: 1) multiplicity of perspectives in defining and understanding equity-focused palliative care; and 2) importance of both wide-ranging financial investment and greater reflection and action on the scope of knowledge required to achieve equity in palliative care.

**Table 2:** Enablers and barriers to equity -focused palliative care

**Enablers**	
Community advocacy	*I think strong community advocacy is probably one of the drivers [of equity in palliative care]. I think the role of Palliative Care SA is another one and then their role is to lobby government and to make sure the community needs are seen, heard and addressed*
Workforce education	*I think education of our workforce that would be not only in understanding what palliative care is, and what the model of care is, but for the general workforce to have better understanding of death literacy, also the needs of different populations.*
The importance of evaluation	*Whatever happens should have an evaluation component and the evaluation to have a policy lens over it. You are not only talking about how you might improve a particular practice, but how might you improve policy to achieve a better outcome.*
The value of new technologies	*I think at end of life can you build up a virtual community for a person who’s dying? There’s lots of apps out there, so that you can actually identify all the people that are in your social network and start to work through them.*
**Barriers**	
Gaps in professional and carer workforce	*I think [barriers are] lack of funding, lack of the provision of palliative care beds, and a lack of workforce. I know that we’re in a particular period now with Covid. It’s just causing so many pressure points in the system and I know that palliative care services just can’t get staff and so as a consequence suffering, burnout of their existing staff.*
Priority setting and fragmented systems of care provision	*We will never see 70% of people dying at home unless we can tackle community care in a really robust kind of way which says you can get the help assistance when you need it, which comes back to equity.*
Funding and resources	*The federal government, by accepting the recommendations in the Royal Commission, haven’t accepted the funding formula recommendations and they haven’t changed the workforce.*
Competition for resources	*It [palliative care] has to compete against other areas of health care, which are much louder. So you’ve got to compete against cardiology, and of course, people want cancer treatments … from a policy point of view, trying to keep palliative care on the agenda means you’ve got to beat that drum even louder because it’s got a lot of noise to breakthrough to be seen.*
Multiple complex barriers	*No one person’s death is the same and their journey and their end of life journey is not the same. So then it really becomes almost every possible barrier that could come up: financial, computer literacy, whether you have family, whether you’re in an aged care facility. It comes to the point that every single possible barrier is another way in which we’re preventing equity of access to palliative care*.

**Table 3:** Examples of evidence gaps and research priorities

Research priority area	Description	Responses
Death and dying	Mapping death literacy and the ‘death journey’	*Research is needed to uncover what death literacy looks like. If we have a greater understanding it helps advocate for family, friends and community, and that could be quite powerful for then impacting on policy change*.
Care models	Community care, compassionate communities, virtual hospitals and the role of nurse practitioners in aged care and co-designing	*I think that there is going to be a huge political driver for this kind of virtual hospital. If you think about how we’ve moved to working from home, having telehealth consultation, you can see that palliative care is likely to be more virtual.* *You’ve got to have the consumer voice not only at the table, but really in designing and co-designing. And my hunch is that there’s not enough of that so if they were really wanting to improve equity, then they would be engaging the very disparate ethnic cultural groups in identifying what are their needs and how may they be facilitated implemented to achieve what the different cultural groups were.*
Economic analyses	The need for baseline data to inform program design and processes	*Seeing a program go from a concept through to funding and implementation requires a series of approval steps. It is important to have robust economic analysis. This also must include the outcomes - what are we getting per input? For example, how do we determine whether to fund one nurse practitioner, or a social worker with 200 boxes of advanced care directives, or four hours of GP time for each GP in the state? How can we support wise investment decisions?*
Access to palliative care	Access to palliative care for different equity groups.	*Priority research areas are country, disability, and complex needs, and understanding that supportive care for other chronic conditions is a problem for palliative care which largely focuses on cancer … We could do models of care in the disability community, which I think is incredibly underdone. Probably there’s some learnings in there too*.

Participants’ views of equity in palliative care included achieving greater access to the full ‘gamut’ of services that aligned with findings from the document review; adequate resource provision for health services, for the palliative care workforce and for improving death literacy; and overcoming structural barriers including the ‘postcode lottery’ of locational disadvantage. One respondent commented:

Access is about greater access to services, the total gamut of service, not just the specialist or generalist ends, but right through the system*.*

Another participant noted:

The ‘post code’ lottery if you happen to live just this side of the line: the great line that divided the regions you would get very different experiences of service delivery because of that*.*

Participants cited a range of South Australian ideas and initiatives to support equitable access. These complemented the strategies that were found in documents and included examples of support for Aboriginal models of palliative care, increasing financial investment in ways which reflect changing needs, and greater support for 24-hour at home funding. One participant stated the increasing attention given to palliative care:

Palliative care is becoming more part of the public and political discourses … It's getting a greater share of the conversation and, so we are seeing investment in different places coming up … It feels like certainly the next five to 10 years will see a significant shift and increase in investment. Probably pretty closely aligned to the aging population as well*.*

Participants also identified enablers and barriers to equity focussed palliative care. Enablers included community advocacy, workforce education, the use of new technologies, and the importance of evaluation.

Barriers included gaps in the professional and carer workforce, fragmented systems of care, lack of funding and /or competition for scarce resources and, for one respondent, the reality of multiple complex barriers for some disadvantaged people. Table 2 demonstrates key enablers and barriers to equity-focused palliative care as reported by interview participants.

Findings about evidence gaps and research priorities for equity-focused palliative care came mainly from interview data. These related to death and dying, care models, economic analyses, workforce and education, and access to palliative care (Table 3).

## Discussion

Equity is at the heart of a public health approach to palliative care. Building public health policies supportive of equity, and strengthening community actions are core components of health promoting palliative care [8]. A critical analysis of palliative care policies is crucial to better understand the policy context and their translation into practice. Key points revealed from our review of palliative care policies and key informant interviews are discussed below:

Firstly, a positive step in considering equity in Australian palliative care policy was evident in documents and was confirmed by our interview participants. The evaluation of the 2010 national policy reported no mention of priority population groups [41]. By comparison, the 2018 national policy included equity and access, with a specific focus on Aboriginal communities and links with Indigenous organisations [30]. This shift of focus was also visible in South Australian 2021–2026 Framework that made a strong commitment to increasing community awareness, understanding and engaging in end-of-life matters; improving access to generalist and specialist services for priority population groups; and enhancing collaboration and coordination [33]. Nevertheless, consideration of equity was mainly confined to reducing access barriers to clinical care for specific population groups rather than addressing systemic barriers and broader advocacy issues relating to social, political and cultural determinants of inequity [46]. Other studies looking at primary health care policies in Australia also report much less attention and action on social determinants of health equity and advocacy at policy level [29, 47]. A policy and political environment supportive of social view of palliative care is essential to enable a move towards equity-focused practice, research and evaluation in palliative care.

Secondly, equity-focused palliative care also requires meaningful community engagement and empowerment and identifying and acting on social determinants of health inequity, or those non-health related issues underpinning poor access and utilisation of services. These include *inter-alia* education, housing, employment, and the legacy of stigma, shame and trauma [48]. A systematic review found that the meaning of and experience of empowerment in people living with advanced life-limiting illness is centred around self-identify (feeling respected and valued) compared to other patient groups which is more around control over their illness [49]. Our study, however, found that current policies and initiatives lack an understanding of different levels of community empowerment in palliative care and identifying specific actions on how to engage and empower communities.

Thirdly, collaboration was mainly seen as vertical collaboration (within health services and organisations or between primary and tertiary services) with minimal evidence of proposed or actual collaboration with sectors outside health. However, strong intersectoral collaboration can lead to increased access to resources, efficiencies, shared risk, and shared learning; recognising that these can deal with issues that would otherwise not be addressed [50]. Although these were acknowledged explicitly or implicitly in the documents, there was no proposed or actual action described to address such factors. Shared leadership, common understanding of the critical role that different organisations play in palliative care and formal agreements with relevant organisations are noted as priority actions that stimulate intersectoral collaboration [51].

Finally, as yet, evaluation frameworks did not use equity, community engagement and social determinants as evaluation domains. Our study identified areas of current gaps in evidence, and research that is needed to inform equity-oriented palliative care policy and practice. Research priorities highlighted by our study respondents align with several raised in a recent systematic review on international palliative care research priorities [24]. This noted critical areas including service models to increase palliative care delivered into the community; continuity of care utilising designated care co-ordinators; training and education, especially of non-palliative care specialists; and inequality of access, including those barriers that are due to social and cultural factors or social determinants of health. Additional research areas were around patient experiences with care, choice and control on place of care and death, death journeys, and the importance of and needs of family carers, including training [24].

Equity-focussed palliative care requires future investment, funding, capacity and commitment at all levels from policy to practice. While expanding the reach of existing palliative care services is critical for improving access and equity, other elements of equity-focused actions including stronger investment in community empowerment to design, implement and evaluate new models of care and community initiatives, and strategies to strengthen intersectoral collaboration and address social determinants of palliative care require further policy and practice consideration.

## Study strengths and limitations

To our knowledge, this is the first study reviewing Australian palliative care policies and initiatives using an equity lens. This is critical to identify gaps and areas that need further consideration. We also interviewed key informants in South Australia to investigate driving forces for equity and the role that research, and evidence can play in informing equity-focused policy and practice. This study is the basis for a future theory-driven policy analysis to look at the history of palliative care policies and changes over time in Australia, nationally and at each jurisdiction, and to undertake comparative studies to explore factors that drive or hinder equity and social determinants of health at policy and practice levels. The study had some limitations. Firstly, due to our limited time and resources, we only included jurisdictional policies from South Australia over the last five years. Furthermore, this study was an exploratory project with a small number of study participants from South Australia. Future studies involving a wider range of actors at policy and practice levels and from different jurisdictions will assist comprehensive analysis of the policy and regulatory environment as well as organisational capacities in developing and implementing equity-oriented palliative care policies in Australia.

## Conclusion

Achieving the goal of equity in palliative care for all is complex and multifaceted. It requires strong commitment and actions at policy and government level but also in clinical practice, workforce planning and capacity building, community engagement and research investment to implement and evaluate public health approaches to palliative care. Further analysis of policy context and driving forces that enable or constrain equity-oriented policy and practice would assist to identify gaps, opportunities and ways to improve quality palliative care for all.

## Supplementary Information

**Additional file 1.**

## Data Availability

Policy and program documents reviewed are publicly available from the Australian government website https://www.health.gov.au/resources?search_api_views_fulltext=Palliative+care The interview datasets generated and analysed during the current study are not publicly available due to confidentiality but are available from the corresponding author on reasonable request.

## Abbreviation

WHO: World Health Organization
