# Supplementary material for: Equity consideration in palliative care policies, programs, and evaluation: an analysis of selected federal and South Australian documents
Source: BMC Palliat Care. 2022 Jun 16;21:109. doi: 10.1186/s12904-022-00997-2 (PMC9202090; doi:10.1186/s12904-022-00997-2)
Supplement: Supplementary file 1 — Additional file 1. [file 12904_2022_997_MOESM1_ESM.docx]

Interview questions

1. To start with, can I ask a broader question on how you define equity in palliative care?

[probes: access to specialist palliative care for under-served population, community based models of palliative care, social determinants of palliative care]

1. How do you think South Australian palliative care policies and initiatives have been able to address inequity?
   1. Examples
   2. Changes over time
   3. Alignment with national directions
2. What do you see as key enablers to equity-oriented policy and practice in South Australia?
3. What do you see as barriers to equity-oriented policy and practice in South Australia?
4. To what extent do you think current policies and programs are informed by evidence and robust research around equity?
5. What do you see as major evidence gaps in South Australia in relation to palliative care policies, models of care and multidisciplinary and intersectoral palliative care services?
6. Can you think of some priority research areas that would help to inform equity-oriented palliative care policy and practice in South Australia? (what would be your 3 first priority?)
